# HIV voluntary testing and perceived risk among female sex workers in the Mekong Delta region of Vietnam

**DOI:** 10.3402/gha.v6i0.20690

**Published:** 2013-07-17

**Authors:** Bach Xuan Tran, Long Thanh Nguyen, Nhung Phuong Nguyen, Huong Thu Thi Phan

**Affiliations:** 1 Institute for Preventive Medicine and Public Health, Hanoi Medical University, Hanoi, Vietnam; 2 Authority of HIV/AIDS Control, Ministry of Health, Hanoi, Vietnam; 3 Division of Epidemiology, Human Genetics and Environmental Sciences, University of Texas Health Science Center at Houston, Houston, TX, USA

**Keywords:** HIV knowledge, perceived risk, HIV testing, female sex workers, Vietnam

## Abstract

**Introduction:**

HIV voluntary counseling and testing (VCT) comprise an effective preventive measure and an entry point to care and support services. We sought to assess VCT uptake and HIV-related knowledge and perceived risk among female sex workers (FSWs) in five provinces of the Mekong Delta region.

**Methods:**

A cross-sectional survey was carried out in 1998 FSWs, including both street-based sex workers (SSWs) and entertainment-based sex workers (ESWs).

**Results:**

High proportions of FSWs were aware that using condoms (94.6%), and clean needles (34.1%) are preventive measures that reduce the risk of HIV transmission. Some FSWs reported avoiding public toilet use (8.6%), physical contacts (16.1%), or sharing meals (10.9%) with people living with HIV/AIDS, and preventing mosquito bites (20.8%). Twenty-nine percent (29.0%) of FSWs perceived themselves as being at risk of HIV infection. Only 32.7% had ever tested for HIV, of whom 54% were voluntary for testing. FSWs who ever injected drugs (OR = 0.03, *p*=0.05), had drug-injecting clients (OR = 0.07, *p*<0.01), and had inconsistent condom use with husbands or lovers (OR = 0.10, *p*=0.01) were less likely to have a voluntary test. Inconsistent condom use with clients (OR = 13.86, *p*<0.01), and receiving HIV information from radio (OR = 13.28, *p*<0.05) and communication campaigns (OR = 6.69, *p*<0.05), increased the likelihood of VCT uptake.

**Conclusion:**

Inadequate knowledge and some misconceptions about HIV transmission routes and preventive measures, low perceived risk of HIV infection, and low VCT uptake were observed among FSWs in the Mekong Delta region. Interventions to improve their knowledge and self-efficacy, reduce risky behaviors, and encourage VCT uptake and early access to health care services are necessary to prevent HIV transmission in this region.

The HIV epidemic in Vietnam comprises many subepidemics across the country and remains concentrated primarily among three high-risk populations: injecting drug users (IDUs), female sex workers (FSWs), and men who have sex with men (MSM) (1). The overlap between unprotected sex and drug injection doubles the risk of HIV infection among FSWs (2). An estimation in 2008 showed that about 3% of FSWs were HIV-positive, and this prevalence varied considerably by provinces (3–5). Only 30% of FSWs reported using condoms consistently with regular partners (5–7). Moreover, the proportion of FSWs who injected drugs was high in metropolitan cities, for example Hanoi (40%) and Ho Chi Minh (13%) (5, 6, 8, 9). Noticeably, those female drug users who also engaged in sex work shared needles more often than male drug users in several provinces (9).

Previous studies have found an association between risky behaviors and inadequate knowledge and low perceived risk of HIV infection among FSWs in various Asian settings (5, 10–14). In China, for instance, FSWs may not use condoms if their clients looked clean or familiar (13). In Turkmenistan, FSWs were not aware of the risk of HIV transmission by sharing needles (12). Wrong perceptions on the modes of spread of HIV were also prevalent among FSWs in Bali, Indonesia. While many of them thought that AIDS can be spread through casual contacts such as shaking hands and eating from the same plate, and that taking antibiotics and traditional medicine could protect them from HIV or sexually transmitted infections (STIs), less than one-fifth perceived their potential exposure to HIV (10). Perceived risk of HIV infection was recognized as a significant predictor of the use of HIV preventive measures (7, 8, 14–18). Low risk perception, therefore, has resulted in low voluntary HIV testing and counseling (VCT) uptake among FSWs in this region. A survey of 6,648 FSWs in India showed that only 8% tested for HIV, and three-quarters of the rest were unwilling to undergo HIV testing in the future (19). A survey conducted in Hanoi, Vietnam, among 400 FSWs also revealed that 55% of the FSWs perceived themselves as being at risk of HIV infection and only 15% were voluntary for a HIV test (14). The wide variability in perceived risk and VCT uptake in different groups and areas suggests the need to characterize environmental and working-related factors influencing the perceptions and behaviors of FSWs.

The Mekong River Delta is located in the south of Vietnam in an area of 40,000 square kilometers (12% of Vietnam's land mass), and is home to over 20% of Vietnam's population. The spread of HIV in this region is primarily driven by the heterosexual route, and sex work is the largest high-risk group. Therefore, to develop effective HIV interventions and encourage earlier access to HIV services, an assessment of a representative sample of FSWs in the region is necessary. The purpose of this study was to evaluate HIV-related knowledge, perceived risk of HIV infection, and VCT uptake among FSWs, and identify their correlated factors in five provinces.

## Methods

### Study design and participant recruitment

A cross-sectional study was conducted in five provinces, including Vinh Long, Ben Tre, Tien Giang, Hau Giang, and Kien Giang, from June 2007 to June 2008. A mapping exercise was conducted prior to implementing the survey following the national guideline (20). We focused on hotpots of sex work in each province. Investigators worked together with outreach program officers, peer educators, and district HIV program managers to identify target groups, data sources, and key informants. Secondary data reviews, field observation, and interviews were conducted to collect and triangulate information about the name and address of each hotpot, and the estimated size of target groups. Following the mapping exercise, we recruited about 400 FSWs in each province who were referred by peer educators or outreach program officers to prevent duplicates in sample selection. This sample size was determined on the basis of a 50% hypothesized HIV testing uptake rate, 5% margin of error, 95% confidence level, and 90% response rate. Consequently, 1998 FSWs were interviewed, including both street-based sex workers (SSWs) and entertainment-based sex workers (ESWs). They were not given any incentive to participate in the study; however, those respondents who seek health care or counseling were referred to relevant service providers. SSWs were defined as FSWs who do not work in formal entertainment establishments, but on the street, in alleys, or in similar places; whereas ESWs include sex workers in restaurants, karaoke, bars, clubs, and massage parlors.

### Measures

The face-to-face interviews are conducted with FSWs. The interviewers’ team is outreach program officers and researchers who were involved in the mapping exercises, and who underwent training and had experience in approaching and interviewing FSWs. A detailed interview procedure with a combination of open and closed questions was developed by the research team. The questionnaire includes demographic characteristics, history of sex work, HIV and STI knowledge, and perceived risk of HIV infection regarding sexual practices and substance use. *HIV-related knowledge* was assessed using nine questions about modes of HIV transmission and preventive measures. These questions have been selected as core indicators for measuring HIV knowledge in the National HIV Monitoring and Evaluation Framework (21). Response options included ‘Yes’, ‘No’, ‘Don't know’, and ‘No answer’. A correct response was scored one, and the other responses were scored zero. The scores for each question were summed to gain a total knowledge score. *HIV perceived risk* was assessed by asking respondents if they thought they were at risk of HIV infection given their current behaviors. *VCT uptake* questions included (1) ever testing for HIV (yes/no), (2) being voluntary for a test (yes/no), and (3) knowing the test result (yes/no).

### Statistical analysis

The *student-t* test and chi-square test were used to examine the differences between means or proportions to describe the characteristics of respondents. *Multivariable logistic regression* was used to assess the association between related factors and dependent variables. The level of significance was set at a *p* value less than 0.05. For the model building, potential predictors included in the full model are demographic characteristics (age, education, and marital status), characteristics of sex work (type of sex work, duration of selling sex, work in other place, and number of clients per month), HIV knowledge, and known risk factors (drug injection, having injecting sexual partners, and inconsistent condom with clients, lovers, and husbands). Using stepwise forward model selection, variables were included in the reduced model when log-likelihood ratio tests give *p*<0.1, and they were excluded at *p* >0.3.

### Ethical considerations

This research project was led by the Vietnam Authority of HIV/AIDS Control. Ethical approval was granted by the Ministry of Health, Vietnam. Respondents were clearly informed about the purposes of the study and gave written informed consent. To protect the identity of respondents, we coded their names in an electronic data set. Original questionnaires were stored in a secured place.

## Results

### Characteristics of participants

The demographic and sex work characteristics of study participants were presented in Table 1. Of 1998 FSWs, 339 (17%) were SSWs and 1,659 (83%) were ESWs. About 24.5 and 4.3% had ever sold sex in other places and overseas, respectively. Mean age was 26.5 at the period of the study; 47.4 and 52.6% completed elementary and secondary school; and 46.8% were single. The mean age of first sexual intercourse and first-time sex work was 18.7 and 23.8, respectively. The mean number of clients was 13.6 in the last month. There were 66.5% respondents who used condoms consistently, and 1.3% reported drug injection.

**Table 1:** Demographic and sex work characteristics among study participants

	Vinh Long (*n*=400)	Ben Tre (*n*=400)	Tien Giang (*n*=400)	Hau Giang (*n*=399)	Kien Giang (*n*=399)	Total (*n*=1,998)	
		
Demographic characteristics	N (%)	N (%)	N (%)	N (%)	N (%)	N (%)	*p*
Education							
Elementary school	153 (38.3)	177 (44.4)	203 (50.8)	206 (51.5)	208 (52.1)	947 (47.4)	<0.01
Secondary school	247 (61.8)	222 (55.6)	197 (49.3)	194 (48.5)	191 (47.9)	1,051 (52.6)	
Resident status							
Living alone	110 (27.5)	93 (23.3)	73 (18.3)	64 (16.2)	107 (26.8)	447 (22.4)	<0.01
Living with family or friend(s)	281 (70.3)	300 (75.0)	325 (81.5)	326 (82.3)	291 (72.9)	1,523 (76.4)	
Unstable	9 (2.3)	7 (1.8)	1 (0.3)	6 (1.5)	1 (0.3)	24 (1.2)	
Marital status							
Single	275 (68.8)	88 (22.0)	132 (33.0)	206 (51.5)	234 (58.7)	935 (46.8)	<0.01
Married or live with partners	47 (11.8)	101 (25.3)	66 (16.5)	45 (11.3)	25 (6.3)	284 (14.2)	
Divorced, separated, or widowed	78 (19.5)	211 (52.8)	202 (50.5)	149 (37.3)	140 (35.1)	780 (39.0)	
	Mean (sd)	Mean (sd)	Mean (sd)	Mean (sd)	Mean (sd)	Mean (sd)	
Age (years)	23.9 (4.7)	30.0 (6.5)	28.2 (8.4)	25.9 (6.1)	24.6 (5.5)	26.5 (6.8)	<0.01
Sex work characteristics							
Age at first sex intercourse	18.1 (1.8)	19.0 (2.2)	18.8 (2.8)	19.5 (2.1)	18.3 (1.6)	18.7 (2.2)	<0.01
Age at first selling sex	21.0 (3.6)	26.8 (6.0)	25.0 (7.3)	23.5 (5.2)	22.7 (4.5)	23.8 (5.8)	<0.01
Length of selling sex (years)	2.9 (2.8)	3.2 (3.4)	3.2 (4.1)	2.3 (2.2)	1.9 (2.3)	2.7 (3.1)	<0.01
Number of clients last month	18.6 (13.7)	11.9 (6.5)	11.6 (8.5)	15 (11.5)	11 (7.6)	13.6 (10.3)	<0.01
Type of sex work	N (%)	N (%)	N (%)	N (%)	N (%)	N (%)	
Street-based sex workers	58 (14.5)	158 (39.5)	67 (16.9)	37 (9.3)	19 (4.8)	339 (17.0)	<0.01
Entertainment-based sex workers	342 (85.5)	242 (60.5)	330 (83.1)	363 (90.8)	380 (95.2)	1,657 (83.0)	
Selling sex in other places	N (%)	N (%)	N (%)	N (%)	N (%)	N (%)	
In other province(s)	125 (31.3)	95 (23.8)	64 (16.1)	112 (28.1)	93 (23.3)	489 (24.5)	<0.01
Overseas	6 (4.8)	1 (1.1)	2 (3.3)	8 (7.2)	4 (4.3)	21 (4.3)	0.30

### HIV-related knowledge among FSWs

Table 2 presents the perception of FSWs on HIV transmission and preventive measures. Almost all respondents were aware of at least one symptom of STI and at least one way to prevent the transmission of HIV. Among these, using condoms was mostly reported (94.6%). However, only 34.1 and 21.0% respectively reported that avoiding unsafe drug injection and multiple sexual partners could reduce the risk of HIV transmission. Some FSWs had misconceptions about HIV prevention and reported that they avoided using public toilets (8.6%), physical contacts with people with HIV/AIDS (16.1%), sharing meals with HIV-positive people (10.9%), and mosquito bites (20.8%). Compared to SSWs, ESWs reported better knowledge on the risk of HIV transmission through needle sharing and fewer misconceptions regarding physical contacts with people with HIV/AIDS.

**Table 2:** HIV-related knowledge, perceived risk, and VCT uptake in SSWs and ESWs

	SSWs (*n*=339)		ESWs (*n*=1,659)		Total		
				
Characteristics	*n*	%	*n*	%	i	%	*p*
HIV-related knowledge							
Knows at least one symptom of STIs	331	97.6	1,632	98.5	1,963	98.4	0.26
Knows measures for preventing HIV transmission							
– Using condoms when having sex	308	94.2	1,518	94.6	1,826	94.6	0.74
– Not sharing needles	87	26.6	571	35.6	658	34.1	<0.01
– Having only one sexual partner	75	22.9	331	20.6	406	21.0	0.35
– Not having sexual contact	3	0.9	34	2.1	37	1.9	0.15
Has wrong perception of modes of HIV transmission							
– Not using public toilets	30	9.2	136	8.5	166	8.6	0.10
– Not exposing self to people living with HIV/AIDS (PLWHA)	77	24.4	229	14.5	306	16.1	<0.01
– Not having meals with PLWHA	42	13.2	166	10.4	208	10.9	0.21
– Avoiding mosquito bites	64	19.6	336	21.1	400	20.8	0.30
Perceived risk of HIV infection							
– No risk	155	46.1	842	51.7	997	50.7	<0.01
– At risk	126	37.5	443	27.2	569	29.0	
– Unknown	55	16.4	334	21.1	399	20.3	
VCT uptake							
– Ever HIV tested	82	24.4	559	34.4	641	32.7	<0.01
– Voluntary for testing	53	64.6	292	52.4	345	54.0	0.11
– Knows the test result	69	84.2	488	87.3	557	86.9	0.43

### Perceived risk of HIV infection and its correlates among FSWs

Twenty-nine percent (29.0%) of respondents perceived that they were at risk of HIV infection, and this was higher in SSWs (37.5%) than in ESWs (27.2%) (Table 2). In logistic regression analysis, the reduced model indicated that a higher likelihood of perceived risk of HIV infection was significantly associated with inconsistent condom use with clients (OR = 4.01; 95% CI = 2.10, 7.66) (Table 3).

**Table 3:** Correlates of perceived risk of HIV infection among FSWs

	Odds ratio (95% CI)	
	
Variables	Univariate	Multivariate
Demographics		
Age (years)	1.03 (1.01; 1.04)***	
Education level (secondary vs. elementary)	1.22 (1.00; 1.49)**	
Marital status (ref = single)		
– Living with husband or partner	2.04 (1.53; 2.70)***	
– Divorced, separated, or widowed	1.41 (1.14; 1.75)***	
Characteristics of sex work		
Type of sex work (ESW vs. SSW)	0.62 (0.49; 0.80)***	0.60 (0.29; 1.26)
Duration of selling sex (years)	1.04 (1.01; 1.07)***	1.05 (0.96; 1.15)
Working in other place or province (yes vs. no)	1.18 (0.95; 1.48)	1.63 (0.78; 3.42)
Number of clients in the last month (>16 clients vs. ≤16 clients)	0.98 (0.79; 1.21)	
Risky behaviors		
Ever injected drug (yes vs. no)	1.94 (0.88; 4.30)	1.15 (0.24; 5.54)
Had IDU clients (yes vs. no)	1.94 (1.14; 3.29)**	0.40 (0.15; 1.08)*
Had IDU husband or lover (yes vs. no)	2.38 (1.17; 4.84)**	3.87 (0.73; 20.43)
Inconsistent condom use with clients	2.73 (2.18; 3.42)***	4.01 (2.10; 7.66)***
Inconsistent condom use with husband	2.14 (1.58; 2.91)***	

*** *p*<0.01

** *p*<0.05

* *p*<0.1.

### VCT uptake among FSW

About one-third of respondents had ever tested for HIV, and this was significantly higher among in ESWs (34.4%) than among SSWs (24.4%). Among those who ever tested, only 54.0% took the test voluntarily, and 86.9% received test results (Table 2). In multivariate analysis, Table 4 shows that duration of sex work (OR = 1.38, *p*=0.04), inconsistent condom use with clients (OR = 13.86, *p*<0.01), receiving HIV information from the radio (OR = 13.28, *p*=0.01), and HIV communication campaigns (OR = 6.69, *p*=0.03) increased the likelihood of taking VCT. Meanwhile, FSWs who had ever injected drug (OR = 0.03, *p*=0.05), had drug-injecting clients (OR = 0.07, *p*<0.01), and used condoms inconsistently with husbands or lovers (OR = 0.10, *p*=0.01) were less likely to take VCT. The perceived risk for HIV infection was significantly associated with a higher likelihood of VCT uptake in the univariate regression but not in the multivariate analysis (Table 4).

**Table 4:** Correlates of VCT uptake among FSWs

	Odds ratio (95% CI)	
	
Variables	Univariate	Multivariate
Demographics		
Age (years)	1.03 (1.01; 1.06)***	
Education level (secondary vs. elementary)	1.05 (0.77; 1.44)	3.41 (0.58; 19.93)
Marital status (ref = single)		
– Living with husband or partner	2.29 (1.39; 3.78)***	
– Divorced, separated, or widowed	1.34 (0.96; 1.88)*	
Characteristics of sex work		
Type of sex work (ESW vs. SSW)	0.60 (0.37; 0.98)**	1.50 (0.20; 11.29)
Duration of selling sex (years)	1.03 (0.98; 1.07)	1.38 (1.01; 1.89)**
Working at other place or province (yes vs. no)	1.09 (0.78; 1.53)	
Number of clients in the last month (>16 clients vs. <16 clients)	0.70 (0.50; 0.98)**	
Risk behaviors		
Ever injected drug (yes vs. no)	1.73 (0.51; 5.79)	0.03 (0.00; 0.94)**
Had injecting drug clients (yes vs. no)	0.6 (0.27; 1.33)	0.07 (0.01; 0.44)***
Had injecting drug husband or lover (yes vs. no)	1.21 (0.35; 4.24)	
Inconsistent condom use with clients	1.07 (0.74; 1.54)	13.86 (2.25; 85.38)***
Inconsistent condom use with husband	0.8 (0.51; 1.28)	0.10 (0.02; 0.51)***
Perceived risk of HIV infection (yes vs. no)	1.44 (1.00; 2.08)**	1.85 (0.29; 11.65)
Sources of HIV information received		
Reading newspaper (yes vs. no)	0.88 (0.65; 1.20)	0.24 (0.03; 1.79)
Listening radio (yes vs. no)	1.70 (1.23; 2.37)***	13.28 (1.69; 104.59)**
Watching Tivi (yes vs. no)	1.57 (0.94; 2.65)*	
HIV communication programs (yes vs. no)	1.1 (0.80; 1.53)	6.69 (1.21; 37.05)**

*** *p*<0.01

** *p*<0.05

* *p*<0.1.

## Discussion

Although almost all respondents know the role of condoms in preventing HIV transmission, a large proportion was not aware of the risks of needle sharing, and had misconceptions on modes of HIV transmission. The perceived risk of HIV infection and VCT uptake were low in both SSWs and ESWs.

FSWs reported condom use more inconsistently with regular clients than with irregular clients, and the most inconsistent use was with husbands or lovers (70.9%). This is in line with findings from previous studies (8, 14, 22, 23). It has been found that given economic pressure, FSWs needed extra money, so they might be less active in negotiating condom use (24, 25). This study also reaffirmed that FSWs engaged in highly risky sexual behavior that put them at risk of contracting HIV or transmitting the disease to male clients. Moreover, we found that selling sex to injecting drug clients caused higher risks of HIV transmission and the spread of HIV between these two at-risk groups. Spouses or lovers of FSWs and IDUs might also be at increased risk of HIV given the inconsistent use of condoms in sexual relations between clients and FSWs.

The perceived risk of HIV infection found in our study was low, which is consistent with other studies among FSWs (10, 14). In our sample, only 29% of FSWs thought that they might be exposed to HIV, which is much lower than findings in Hanoi (55%) and in 2011 HIV surveillance data that was gathered from 12 provinces (43.8%). This study is also alarming regarding the low accessibility to HIV testing among FSWs in the Mekong Delta region. VCT is considered an entry point for other interventions on HIV/AIDS. Significant progress has been made in Vietnam to scale up HIV testing in recent years, with the number of VCT sites increasing from 157 sites in 2005 to 317 sites in 2011. Along with the rapid expansion of VCT services, communication, education, and outreach programs need to target FSWs with low accessibility, for example those with drug use, inconsistent condom use, having sex partners who are IDUs, and working in restaurants. The association between perceived risk of HIV infection and HIV testing was significant in univariate analysis, which is similar to a study in India by Dandona et al. (19). Those FSWs who perceived their risk of HIV infection were more likely to take the VCT, suggesting the importance of improving the self-efficacy of FSWs in recognizing modes of HIV transmission and risky behaviors. Findings of this study also inform the selection of channels for HIV information, communication, and education campaigns targeting FSWs. Communication campaigns targeting FSWs can improve the uptake of VCT substantially (26, 27). As for mass media, both radio and TV programs were good sources of HIV information that were associated with VCT uptake among FSWs; meanwhile, newspapers showed limited impact.

Our study has some limitations. First, participants may have underreported risk behaviors due to participants’ perceived risks, motivation, and cultural ideas and norms about disclosing sensitive personal details (6, 13). Second, causal inferences about predictors of HIV testing might not be confirmed given the cross-sectional nature of the study design. Nonetheless, this is the very first large-scale survey among FSWs in the Mekong Delta region. Findings of this study provide evidence about the need to focus on more comprehensive information, education, and communication campaigns for FSWs. Many campaigns show positive impacts on changing HIV-related behavior in developing countries, and these can be good models to be adopted in Vietnam (28). In the Mekong Delta region, priority interventions should focus on providing adequate knowledge on transmission routes, ways of prevention, and eliminating misconceptions regarding HIV transmission routes. In addition, the low level of education and the different working environment between SSWs and ESWs should be considered to choose appropriate channels of informing and meeting the needs of each subgroup of FSWs.

## Conclusion

There were inadequate knowledge and some misconceptions about HIV transmission routes and preventive measures, low perceived risk of HIV infection, and low VCT uptake among FSWs in the Mekong Delta region. Interventions to improve their knowledge and self-efficacy, reduce risky behaviors, and encourage VCT uptake and early access to health care services are necessary to prevent HIV transmission in this region.
